# Structural Advances in Voltage-Gated Sodium Channels

**DOI:** 10.3389/fphar.2022.908867

**Published:** 2022-06-03

**Authors:** Daohua Jiang, Jiangtao Zhang, Zhanyi Xia

**Affiliations:** ^1^ Laboratory of Soft Matter Physics, Institute of Physics, Chinese Academy of Sciences, Beijing, China; ^2^ University of Chinese Academy of Sciences, Beijing, China; ^3^ Key Laboratory of Molecular Biophysics of MOE, College of Life Science and Technology, Huazhong University of Science and Technology, Wuhan, China

**Keywords:** voltage-gated sodium channel, cryo-EM, pharmocology, gating mechanism, drug modulation mechanism

## Abstract

Voltage-gated sodium (Na_V_) channels are responsible for the rapid rising-phase of action potentials in excitable cells. Over 1,000 mutations in Na_V_ channels are associated with human diseases including epilepsy, periodic paralysis, arrhythmias and pain disorders. Natural toxins and clinically-used small-molecule drugs bind to Na_V_ channels and modulate their functions. Recent advances from cryo-electron microscopy (cryo-EM) structures of Na_V_ channels reveal invaluable insights into the architecture, activation, fast inactivation, electromechanical coupling, ligand modulation and pharmacology of eukaryotic Na_V_ channels. These structural analyses not only demonstrate molecular mechanisms for Na_V_ channel structure and function, but also provide atomic level templates for rational development of potential subtype-selective therapeutics. In this review, we summarize recent structural advances of eukaryotic Na_V_ channels, highlighting the structural features of eukaryotic Na_V_ channels as well as distinct modulation mechanisms by a wide range of modulators from natural toxins to synthetic small-molecules.

## Introduction

Voltage-gated sodium (Na_V_) channels are a family of integrated membrane proteins, which selectively conduct sodium ions across cell membrane in response to depolarizing stimuli (Catterall, 2000; Hille, 2001). The primary function of Na_V_ channels was related to the generation of action potentials (Hodgkin And Huxley, 1952a; Hodgkin And Huxley, 1952b). Subsequently, the voltage-dependent activation, sodium selectivity, fast inactivation and components of Na_V_ channels were characterized by extensive biophysical and biochemical studies (Armstrong and Bezanilla, 1973; Tamkun and Catterall, 1981; Weigele and Barchi, 1982; Hartshorne and Catterall, 1984; Stühmer et al., 1987). Since the first Na_V_ channel gene was cloned by Noda in 1984 (Noda et al., 1984), nine highly conserved Na_V_ channel subtypes (Na_V_1.1- Na_V_1.9) in humans have been identified (Catterall et al., 2005). These channels have specific tissue-expression patterns. Na_V_1.1, Na_V_1.2 and Na_V_1.3 are mainly expressed in the central nervous system (CNS), which are crucial for nerve excitability. Hundreds of mutations in these channels cause inherited epilepsy and other form diseases of hyperexcitability (Meisler and Kearney, 2005; Catterall et al., 2008; Catterall, 2014; Huang et al., 2017). Phenytoin, lamotrigine, and carbamazepine are clinical drugs for treatment of epilepsy that act as Na_V_ channel blockers (Zuliani et al., 2012; Catterall, 2014). Na_V_1.4 and Na_V_1.5 are predominantly expressed in skeletal muscle and cardiomyocytes, respectively. Malfunction of these two Na_V_ channels are associated with periodic paralysis, myotonia and cardiac arrhythmias (Sokolov et al., 2007; Catterall et al., 2020a). Class I anti-arrhythmic drugs, which block Na_V_1.5 to remove the abnormal component, are broadly used for treating arrhythmias. Based on the rate of binding and unbinding, the Class I anti-arrhythmic drugs are divided into three subclasses: IA (*e.g.*, quinidine), IB (*e.g.*, lidocaine) and IC (*e.g.*, flecainide) (Kowey, 1998; Huang et al., 2020). Na_V_1.6 is widely distributed in both CNS and the peripheral nervous system (PNS). The unique features of Na_V_1.6 such as generating persistent current and resurgent current, contribute to neuronal excitability and repetitive neuronal firing (Raman and Bean, 1997; Raman et al., 1997). Mutations in Na_V_1.6 are related to epilepsy, ataxia and dystonia (O'Brien and Meisler, 2013). Na_V_1.7, Na_V_1.8 and Na_V_1.9 are highly expressed in PNS, which are closely related to pain perception (Bennett et al., 2019; Dib-Hajj and Waxman, 2019). Na_V_1.7 was also reported to be essential for odour sensation (Weiss et al., 2011). Many programs have been established in searching for Na_V_1.7 and Na_V_1.8 selective inhibitors as potential analgesics (Kingwell, 2019; Alsaloum et al., 2020). In addition, a wide range of natural toxins from animal or plant venoms target the Na_V_ channels and modulate channel functions by binding in at least six distinct receptor sites (Catterall et al., 2007).

**FIGURE 1:**
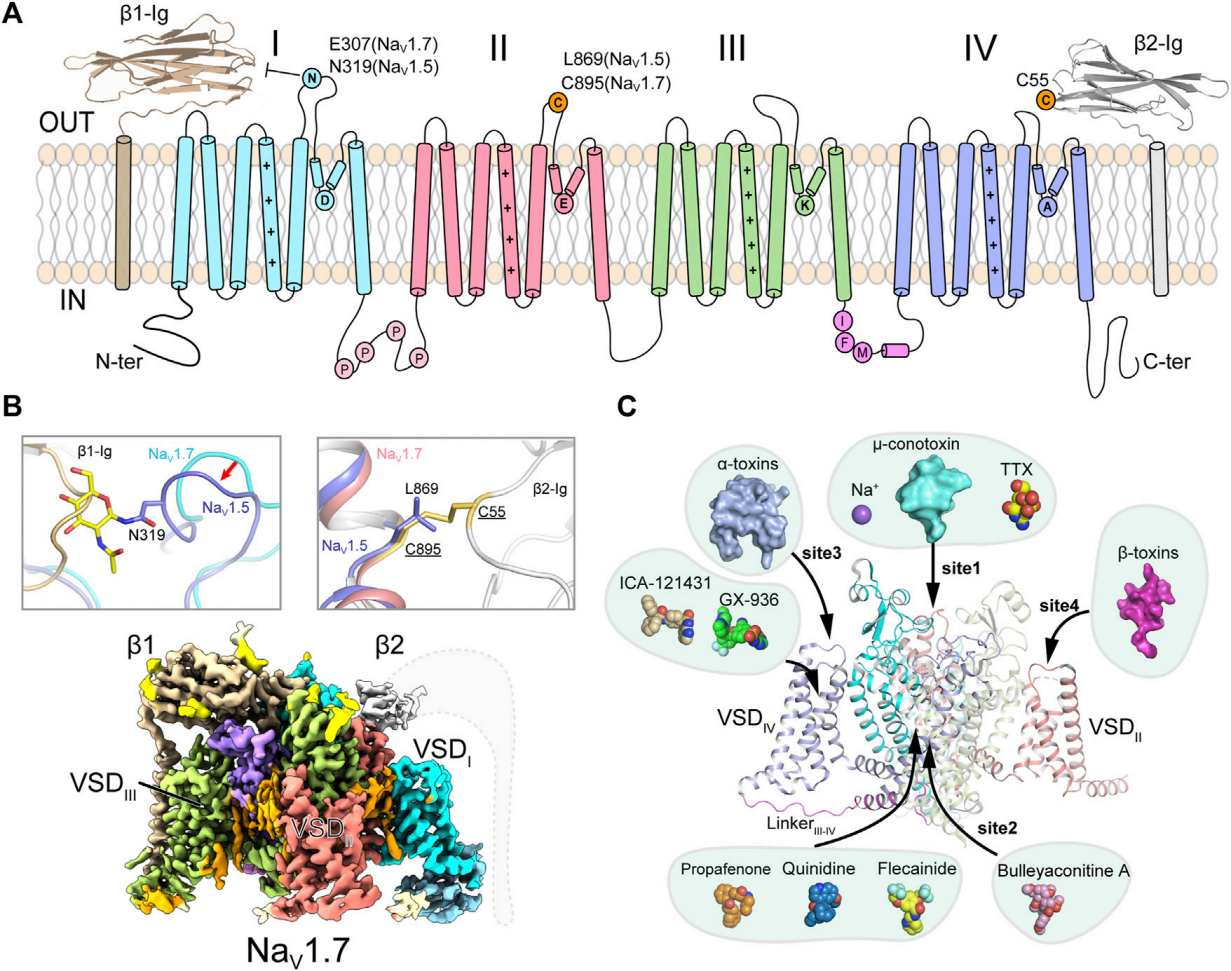
Topology and architecture of mammalian Na_V_ channels. **(A)** Topology of mammalian Na_V_ channels. D_I_, D_II_, D_III_, IFM-motif and D_IV_ of α-subunit, β1 and β2 subunit are colored in cyan, red, green, purple, blue, brown and gray, respectively. A unique N-glycosylation site in Na_V_1.5 and the cysteine residue for linking β2 are highlighted. P represents phosphorylation sites. **(B)** Subunits assembly of mammalian Na_V_ channels. A sugar moiety of N319 glycosylation and L869 block the binding of β1 and β2 to Na_V_1.5 (PDB code: 6UZ3), respectively (Upper panels). Cryo-EM map of human Na_V_1.7/β1/β2 (lower panel, EMDB code: EMD-33292). Dashed lines indicate the invisible region of β2 subunit. **(C)** Multiple ligand binding sites in mammalian Na_V_ channel α-subunit. Sodium ion and small-molecule modulators shown in spheres, polypeptide toxins shown in surface.

Further studies revealed that Na_V_ channels are widely distributed in eukaryotes, as well as in prokaryotes (Koishi et al., 2004; Ren et al., 2001) and marine unicellular phytoplankton (Helliwell et al., 2020; Helliwell et al., 2019), highlighting the evolutionary conservation of Na_V_ channels. The metazoan Na_V_ channels are composed of a large pore-forming α-subunit and one or two auxiliary β-subunits (β1-β4) (Figure 1A) (Catterall, 2000; Hartshorne and Catterall, 1984; Isom et al., 1994; O'Malley and Isom, 2015). The α-subunit consists of 24-transmembrane helices, which are divided into four homologous domains (D_I_-D_IV_). The four domains are connected by intracellular loops. The loop between D_III_ and D_IV_ contains a highly conserved fast inactivation gate, which mediates the fast inactivation of Na_V_ channels (West et al., 1992; Goldin, 2003). The first four transmembrane segments (S1-S4) form the voltage-sensing domain (VSD), the S5 and S6 form the pore module (PM). Distinct from the metazoan Na_V_ channels, Na_V_ channels from bacteria and the unicellular phytoplankton are formed by four identical protomers in a homotetrameric organization (Ren et al., 2001; Helliwell et al., 2020). In 2001, Sato pioneered the structural study of Na_V_ channels reporting a ∼19 Å cryo-electron microscopy (cryo-EM) map of Na_V_ channel from the electrical eel (Sato et al., 2001). However, the low-resolution map did not permit model building of the Na_V_ channel. The crystal structure of voltage-gated potassium (K_V_) channel also provided useful homologous template for Na_V_ channels (Long et al., 2005). Since 2011, Catterall lab and other groups reported high-resolution crystal structures of prokaryotic Na_V_ (BacNa_V_) channels (Payandeh et al., 2011; Payandeh et al., 2012; Zhang et al., 2012; Lenaeus et al., 2017; Sula et al., 2017; Wisedchaisri et al., 2019), which revealed structural basis for bacterial Na_V_ channel architecture, activation and inactivation, gating, ion conductance and selectivity at atomic level. These bacterial Na_V_ channels had also been used as templates to investigate the structural pharmacology of human Na_V_ channels (Bagnéris et al., 2014; Boiteux et al., 2014; Ahuja et al., 2015; Tang et al., 2016; Gamal El-Din et al., 2018; Wisedchaisri et al., 2021). Recent advances from cryo-EM structures of the eukaryotic Na_V_ channel Na_V_Pas (Shen et al., 2017; Shen et al., 2018; Clairfeuille et al., 2019), Na_V_1.4 (Yan et al., 2017; Pan et al., 2018), Na_V_1.7 (Shen et al., 2019), Na_V_1.2 (Pan et al., 2019), Na_V_1.5 (Jiang et al., 2020; Jiang et al., 2021a; Li et al., 2021a; Li et al., 2021b; Jiang et al., 2021c), Na_V_1.1 (Pan et al., 2021) and Na_V_1.3 (Li et al., 2022) reveal detailed structural basis for the eukaryotic Na_V_ channel subunits assembly, fast inactivation, modulation by natural animal toxins, and pore blockade by anti-arrhythmic drugs (Figures 1B,C). In this review, we highlight the structural features of the eukaryotic Na_V_ channels and the distinct molecular mechanisms for Na_V_ channel modulation by natural toxins and clinical drugs.

### Assembly of Voltage-Gated Sodium Channels

The homotetrameric bacterial Na_V_Ab had been an ideal model for investigating molecular mechanisms of Na_V_ channel activation, inactivation, gating, electromechanical coupling and mutation induced channelopathies (Payandeh et al., 2011; Payandeh et al., 2012; Lenaeus et al., 2017; Jiang et al., 2018; Wisedchaisri et al., 2019) (Reviewed in Ref (Catterall et al., 2020b)). However, there are significant structural and functional differences between the homotetrameric BacNa_V_ channels and the asymmetric eukaryotic Na_V_ channels. The eukaryotic Na_V_ channels withheld their detailed structural features owing to their extremely low yield of recombinant expression and sample heterogeneity. In 2017, Nieng Yan lab reported the ground breaking high-resolution cryo-EM structure of a eukaryotic Na_V_ channel Na_V_Pas from the American cockroach (Shen et al., 2017). Although Na_V_Pas was found to be non-functional in heterologous expression system and lack of the conserved fast inactivation gate, the Na_V_Pas structure does provide important insights into the architecture of Na_V_ channel α-subunit, four VSDs in distinct conformations and the asymmetric selectivity filter (SF). In addition, the structure proved that human Na_V_ channels could possibly be resolved to high-resolution by the advanced cryo-EM technique. After extensive efforts had been conducted to improve the sample quality (Jiang et al., 2021b; Shen et al., 2021), cryo-EM structures of the electrical eel and human Na_V_1.4-β1, human Na_V_1.7-β1-β2, human Na_V_1.2-β2, rat and human Na_V_1.5, human Na_V_1.1-β4 and human Na_V_1.3-β1-β2 were subsequently reported at 3–4 Å resolution (Yan et al., 2017; Pan et al., 2018; Pan et al., 2019; Shen et al., 2019; Jiang et al., 2020; Li et al., 2021b; Pan et al., 2021; Li et al., 2022). These structures reveal detailed insights into the assembly of Na_V_ channel α- and β-subunits, allosteric inhibition mechanism of the fast inactivation and multiple ligand binding sites (Figures 1B,C).

These high-resolution structures suggest that the overall structures of the Na_V_ channels are structurally conserved. The transmembrane core-regions closely resemble the bacterial Na_V_ channels, which are formed similarly in a domain-swapped manner, e.g., VSD and PM of one domain are separated by a S4S5 linker helix, thus VSD of one domain closely engages PM from the other domain. Distinct from the BacNa_V_ channels, the metazoan Na_V_ channels possess large extracellular loops (ECLs) between S5 and S6 helices of each domain. Multiple glycosylation sites on the ECLs were visualized from these structures, which are important for the channel maturation and modulation. On the cytoplasmic side, the N-terminal domain (NTD), long loops of D_I_-D_II_ and D_II_-D_III_, and the C-terminal domain (CTD) were invisible to cryo-EM, which are probably due to mobility of these regions. These intracellular regions were reported to be essential for Na_V_ channel function and modulation (West et al., 1991; Mantegazza et al., 2001; Scheuer, 2011; Clatot et al., 2012). However, it’s challenging to capture a snapshot of the transient modulation state such as phosphorylation. The relative short loop between D_III_-D_IV_ of the mammalian Na_V_ channels, which contains the fast inactivation particle Ile-Phe-Met (IFM) motif, were all clearly resolved in a nearly identical conformation (Pan et al., 2018; Pan et al., 2019; Shen et al., 2019; Jiang et al., 2020; Pan et al., 2021). The IFM-motif binds to a hydrophobic pocket adjacent to the intracellular activation gate, rather than directly blocking the activation gate, elucidating a unique local allosteric inhibition mechanism for the fast inactivation.

The β-subunits (β1-β4) bind to the α-subunit and regulate the Na_V_ channel properties such as cell surface expression, voltage-dependent activation and gating kinetics (Hartshorne and Catterall, 1984; Isom et al., 1994; O'Malley and Isom, 2015; Vijayaragavan et al., 2001). The four β-subunits share conserved topology of an extracellular immunoglobulin-like domain and a single transmembrane helix (Das et al., 2016; Gilchrist et al., 2013; Namadurai et al., 2014). The Na_V_1.4-β1, Na_V_1.7-β1-β2, Na_V_1.2-β2, Na_V_1.1-β4 and Na_V_1.3-β1-β2 complex structures confirmed that the β1 noncovalently binds to the α-subunit, whereas β2 or β4 is linked to the α-subunit *via* a disulfide bond (Figures 1A,B). Surprisingly, the rat and human Na_V_1.5 structures showed that the β1 binding is blocked by a sugar moiety of a unique glycosylation modification in the ECL_I_, and the cysteine for linking β2 or β4 is substituted by a Leu869 in Na_V_1.5 or an Ala822 in Na_V_1.8 (Figure 1B) (Jiang et al., 2020; Li et al., 2021a). These structural observations demonstrate that the β subunits bind weakly to Na_V_1.5, which is in agreement with previous biophysical reports that the β subunits have negligible effect on the kinetics or voltage-dependence of Na_V_1.5 (Makita et al., 1994; Qu et al., 1995). However, pathogenic mutations in the Na_V_ β-subunits were reported to be associated with cardiac arrhythmias (Ruan et al., 2009; Watanabe et al., 2009; Olesen et al., 2011). These results indicate that the β-subunits might be important for Na_V_1.5 folding or trafficking to the plasma membrane, rather than modifying Na_V_1.5 functional properties. It has also been reported that the β-subunits play other physiological functions unrelated to Na_V_1.5, such as cell adhesion in the cardiomyocytes (Patino et al., 2011; Namadurai et al., 2015).

### Ion Path of Na_V_ Channels

**FIGURE 2:**
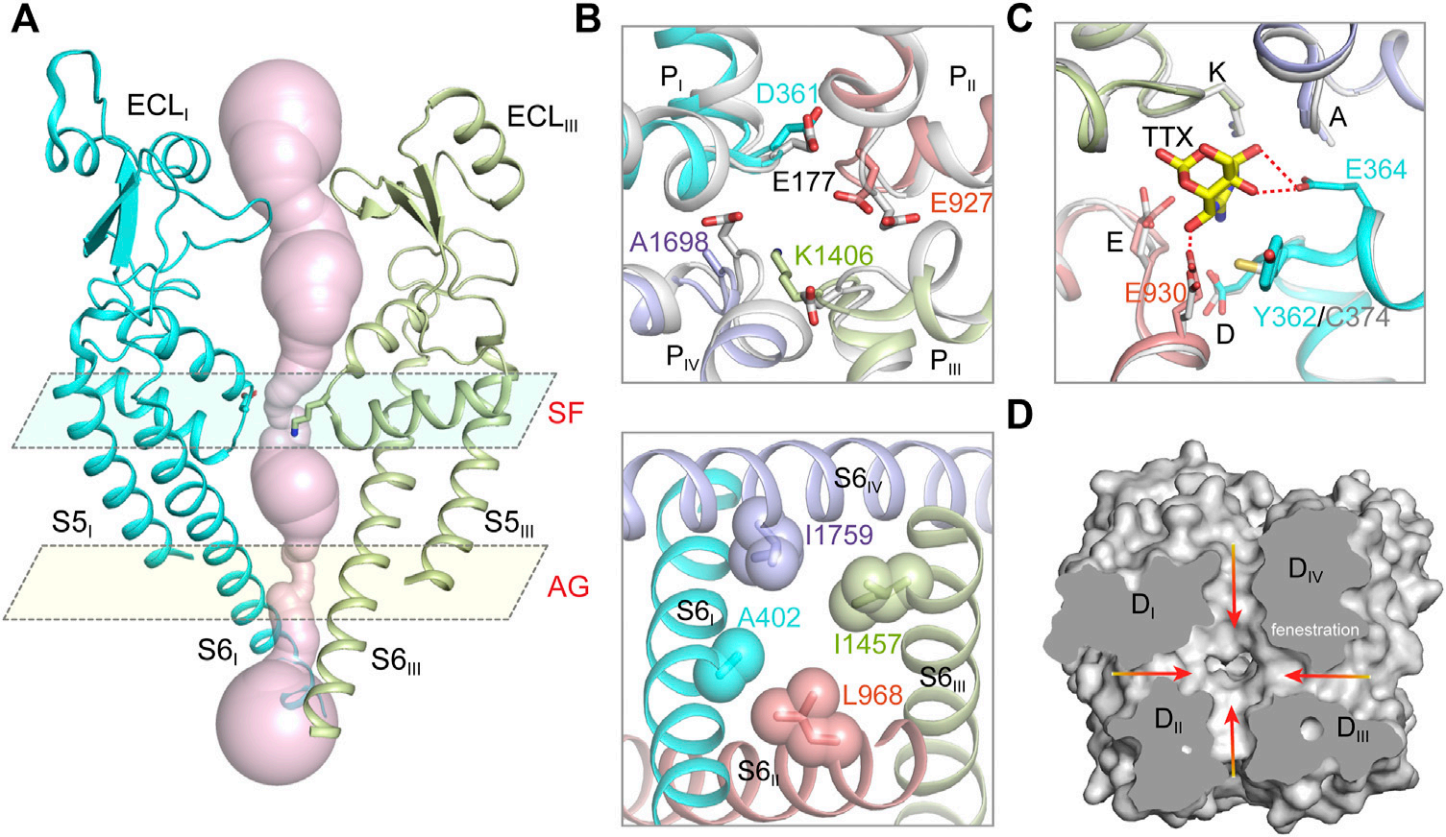
The ion path of mammalian Na_V_ channels. **(A)** The pore radii of human Na_V_1.7 (PDB code: 7XM9) calculated by HOLE. Only two opposing subunits shown for clarity. The constriction sites of selectivity filter (SF) and intracellular activation gate (AG) are highlighted. **(B)** A close-up view of the SF (top panel) and AG (bottom panel) of human Na_V_1.7 (PDB code: 7XM9). The SF of Na_V_1.7 was aligned with the SF of Na_V_Ab (gray, PDB code: 3RVY). The side-chains of key residues in the SF and the AG are depicted in sticks. **(C)** The TTX binding site in Na_V_1.7 (PDB code: 6J8I). The TTX-insensitive channel Na_V_1.5 (PDB code: 6UZ3) shown in gray. **(D)** Fenestrations of human Na_V_1.7 (PDB code: 7XM9). Red arrows indicate potential access for lipids or pore-blockers.

Hille tested the sodium selectivity of Na_V_ channels and estimated a ∼3.0 Å by ∼5.0 Å constriction site at the extracellular side (Hille, 1971a; b; 1972; 1975). Point-mutagenesis studies revealed that an Asp in D_I_, Glu in D_II_, Lys in D_III_ and Ala in D_IV_, often termed the DEKA-locus, are responsible for the sodium selectivity (Favre et al., 1996; Sun et al., 1997). The crystal structure of the bacterial Na_V_Ab provides the first atomic model for investigating the molecular mechanism of sodium selectivity (Payandeh et al., 2011). At the center of Na_V_ channels, four pore-loops (P-loop) and S6 helices form an ion conductance path across the membrane. The ion path consists of a narrow extracellular constriction site termed selectivity filter (SF), a large central cavity (CC) and a size-variable intracellular activation gate (AG) (Figure 2A). Unlike the asymmetric DEKA-locus of the metazoan Na_V_ channels, four identical P-loops (TLESWSM) of Na_V_Ab constitute the SF with diameter of ∼4.6 Å by ∼4.6 Å, close to Hille’s estimation (Hille, 1975; Payandeh et al., 2011). The SF of Na_V_Ab is significantly wider than that of potassium channels, suggesting that sodium may pass through the SF in partially hydrated form (Doyle et al., 1998; Hille, 1971a; Payandeh et al., 2011; Yellen, 2002). The four Glu at +3 position of the SF loop determine sodium selectivity, which form a high-field strength site to coordinate sodium ions (Figure 2B). Substitution of the TLESWSM with TLDSWDD converts the sodium channel to a highly calcium selective channel (Yue et al., 2002; Tang et al., 2014), suggesting that the ion path of sodium and calcium channels are closely related. Crystal structure of the chimeric calcium channel Ca_V_Ab revealed high affinity binding sites for Ca^2+^ supporting the stepwise “knock-off” permeation mechanism, which are not observed in the BacNa_V_ channel structures (Payandeh et al., 2011; Zhang et al., 2012; Tang et al., 2014). These observations suggested that the Na_V_ and Ca_V_ channel are evolutionarily highly-related, the subtle compositional differences in the SFs discriminate Na^+^ and Ca^2+^ ions. Based on the BacNa_V_ structures and molecular dynamics simulation studies, potential models had been proposed to explain the sodium selectivity (Corry and Thomas, 2012; Chakrabarti et al., 2013; Naylor et al., 2016). However, these possible mechanisms may not be fully applicable to the metazoan Na_V_ channels, because the asymmetric DEKA-locus is absent in the BacNa_V_ channels.

The cryo-EM structures of metazoan Na_V_ channels revealed that the SF is comparable to that of the BacNa_V_ structures (Jiang et al., 2020; Pan et al., 2019; Pan et al., 2018; Shen et al., 2019; Shen et al., 2017). The Asp in D_I_ and Glu in D_II_ of the DEKA-locus provide the high-field strength site similar to the BacNa_V_ channels (Figure 2B), which coordinates and facilitates partially dehydration of sodium ions (Payandeh et al., 2011; Chakrabarti et al., 2013). The Lys in D_III_ of the DEKA-locus was consistently found pointing its sidechain deep inside the SF and narrowing the SF in one dimension, which meets Hille’s estimation (Hille, 1971b, 1975; Jiang et al., 2020). A positively-charged Lys embedded in the SF would likely block the ion permeation rather than conduct. Considering pKa value of Lys-NH2 can be dramatically affected by its interacting chemical environments (Isom et al., 2011), one possibility is that the pKa of the Lys is significantly decreased and its positive charge is delocalized by nearby carbonyl groups (Jiang et al., 2020). In this case, the neutralized Lys may serve as an energetically favorable coordinating ligand for Na^+^ or Li^+^, whereas the larger alkali cations like K^+^, Rb^+^ and Cs^+^ are energetically unfavorable.

The tetrodotoxin (TTX) from puffer fish is a highly Na_V_ channel specific and highly lethal neurotoxin, which was initially used to discriminate Na^+^ and K^+^ currents in the squid giant axons and to map the location of the SF (Kao, 1966; Noda et al., 1989). Based on the binding affinity, Na_V_1.1, Na_V_1.2, Na_V_1.3, Na_V_1.4, Na_V_1.6 and Na_V_1.7 are classified as TTX-sensitive channels with TTX affinity at nanomolar level; Na_V_1.5, Na_V_1.8 and Na_V_1.9 are TTX-insensitive channels with micromolar affinity. A Tyr or Phe residue located in the D_I_ P-loop was found as the key determinant for the TTX sensitivity, this residue is substituted by a Cys or Ser in the TTX-insensitive Na_V_ channels (Sivilotti et al., 1997; Sunami et al., 2000). The detailed binding site for TTX and another guanidinium neurotoxin Saxitoxin (STX), termed site-1, was depicted by the high-resolution cryo-EM complex structures of Na_V_Pas and Na_V_1.7 (Shen et al., 2018; Shen et al., 2019). The positively-charged TTX or STX forms extensive electrostatic interactions with the negatively-charged vestibule of the Na_V_ channels, that physically blocks ions entering the SF. The Tyr on D_I_ P-loop of Na_V_1.7 forms an additional π-cation interaction with the TTX or STX, which is absent in Na_V_1.5 (Figure 2C). Consequently, the affinity of TTX for Na_V_1.7 is over 500-fold higher than that for Na_V_1.5. These structures provide an excellent example showing that a single residue at the receptor site could confer isoform selectivity. Recently, guanidinium neurotoxin analogues ST-2262 and ST-2530 were discovered as potent and selective Na_V_1.7 inhibitors (Pajouhesh et al., 2020), suggesting that the TTX receptor site is a potential therapeutic site. Because the blockade of the guanidinium neurotoxin is state-independent, high affinity and isoform selectivity is critical to minimize potential side effects. The structures of human Na_V_ channel complexed with the guanidinium neurotoxins should facilitate rational development of isoform selective candidate drugs.

### The Central Cavity of Na_V_ Channels

**FIGURE 3:**
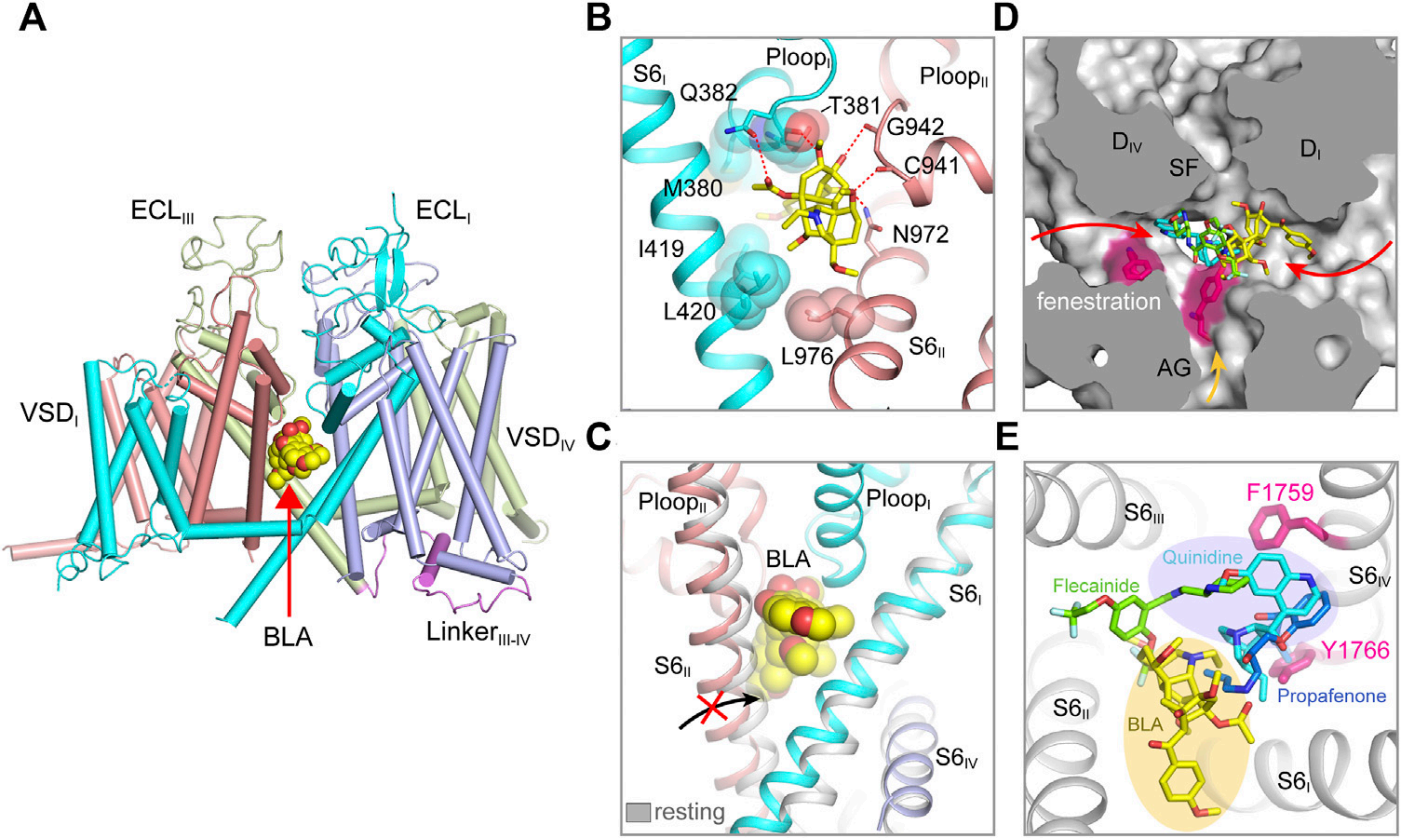
Pore modulators of mammalian Na_V_ channels. **(A)** The binding site of site-2 neurotoxin BLA in Na_V_1.3 (PDB code: 7W77). BLA is depicted in spheres. **(B)** A close-up view of the BLA binding site. Key interacting residues are shown side chains in sticks. BLA is shown in sticks. **(C)** BLA binding prevents S6_II_ helix shifting to the resting state. **(D)** Superposition of Na_V_ structure bound BLA (PDB code: 7W77), Flecainide (PDB code: 6UZ0), Propafenone (PDB code: 7FBS) and Quinidine (PDB code: 6LQA) in the pore. Arrows indicate possible access path for the drugs. **(E)** Top-down view of the BLA binding site and the antiarrhythmic drug binding sites from panel **(D)**.

The bulky central cavity of Na_V_ channels connects the extracellular SF and the intracellular activation gate, but also opens to four fenestrations formed by adjacent PMs (Figure 2D). Lipids or small-molecule drugs access the central cavity through these connecting tunnels to modulate channel properties. The central cavity accommodates multiple receptor sites for site-2 neurotoxins (Deuis et al., 2017), local anesthetic, anti-epilepsy and antiarrhythmic drugs (Hille, 1977; Hondeghem and Katzung, 1984). Batrachotoxin, veratridine and aconitine are natural alkaloids, which modulate the voltage-dependent activation, inactivation and ion selectivity of Na_V_ channels (Catterall et al., 2007; Quandt and Narahashi, 1982; Ulbricht, 1969). The site-2 neurotoxins usually activate Na_V_ channels at hyperpolarized membrane potential or increase the open probability, thus they are considered as Na_V_ channel activators (Deuis et al., 2017). Bulleyaconitine A (BLA), the active substance of the traditional herb *Aconitum bulleyanum* plant (Tang et al., 1986; Wang et al., 2007), exhibits strong inhibition of Na_V_ channels in a use-dependent manner. The complex structure of human Na_V_1.3-β1-β2 with BLA reveals a binding site for the site-2 neurotoxin (Li et al., 2022). BLA binds tightly to the central cavity of Na_V_1.3 *via* multiple polar and non-polar interactions with P-loops and S6 helices of D_I_ and D_II_ (Figure 3A). The BLA engages V416 and L420 on D_I_-S6 helix, N972 and L976 on D_II_-S6 helix (Figure 3B), which are in line with the biophysical studies that these residues are important for batrachotoxin, grayanotoxin or veratridine binding (Wang and Wang, 1998; Ishii et al., 1999; Wang et al., 2000; Wang et al., 2001). However, D_III_-S6 and D_IV_-S6 helices, which were reported to be involved in batrachotoxin binding (Wang and Wang, 1999; Deuis et al., 2017), have no direct contact with BLA in the Na_V_1.3-BLA complex structure. These results suggest that the site-2 neurotoxins may bind to overlapping but not identical receptor sites in the central cavity. The binding of BLA partially blocks the ion path, which causes the strong inhibitory effect on Na_V_1.3. The weak activation potency of BLA probably because BLA sticks into the D_I_-D_II_ fenestration, which prevents the closure of the pore-lining S6 helices of D_I_ and D_II_ during state transition (Figure 3C). Owing to the shape and chemistry differences, site-2 neurotoxins with strong activation potency such as batrachotoxin and veratridine likely bind in the central cavity partially overlapping with the BLA site, which stabilize the pore in open conformation and cause less pore blockade.

The structures of Na_V_1.5 in complex with antiarrhythmic drugs flecainide, propafenone and quinidine reveal antiarrhythmic drugs binding sites in the central cavity (Jiang et al., 2021a; Jiang et al., 2020; Li et al., 2021a), which are distinct from the BLA binding site (Figures 3D,E). All three drugs block Na_V_1.5 in use-dependent manner. The Class IC drug flecainide binds in the central cavity close to D_II_-D_III_ fenestration. Its positively-charged piperidine nitrogen lies beneath the SF, which prevents the exit of Na^+^ from the SF. Meanwhile, the hydrophobic side of the piperidine ring faces the sidechain of Phe1759 (rat Na_V_1.5), the key residue for antiarrhythmic and local anesthetic drugs binding (Ragsdale et al., 1994). Mutation of this Phe1759 to Ala significantly drops the affinity of flecainide (Liu et al., 2003; Ragsdale et al., 1996; Wang et al., 2003). One of the two hydrophobic trifluoromethoxy tails of flecainide is inserted into the D_II_-D_III_ fenestration, which suggests that flecainide may access the binding sites through the fenestrations (Gamal El-Din et al., 2018; Nguyen et al., 2019). Compared to class IA (e.g., quinidine) and IB (e.g., lidocaine) drugs, flecainide is larger in size and more hydrophobic, the higher affinity and slower binding kinetics of flecainide and other class IC drugs likely reflect their stronger interaction with Na_V_1.5. The structure of human Na_V_1.5-quinidine reveals a quinidine binding site which is also located in the central cavity (Li et al., 2021a). Quinidine lies under the SF and physically blocks the ion path. The quinolone ring of quinidine overlaps with the piperidine ring of flecainide (Figure 3D). Unlike the flecainide binding without causing obvious local conformational changes, the quinidine binding was reported to induce the side chain rotation of Tyr1767 (human Na_V_1.5) and a slightly smaller activation gate compared to the human Na_V_1.5 structure with a Glu1784Lys mutation (Li et al., 2021a; Pan et al., 2021). However, similar “up” and “down” conformations of the Tyr1755 at the equivalent position were also observed in the Na_V_1.7 structures (Shen et al., 2019). In addition, the side chain rotation of Tyr1767 could also be affected by the pathogenic Glu1784Lys mutation, which alters the Na_V_1.5 gating by significantly shifting the voltage-dependent activation toward depolarized membrane potential and the voltage-dependent inactivation toward hyperpolarized membrane potential (Makita et al., 2008).

Class IC drug propafenone preferentially blocks open Na_V_1.5 (Kohlhardt and Fichtner, 1988). The open state structure of Na_V_1.5 was achieved by importing the IFM/QQQ mutations (termed Na_V_1.5/QQQ) to remove the fast inactivation gate, also with the help of propafenone blocking the constant opening activity of the Na_V_1.5/QQQ during protein expression (Jiang et al., 2021a). The Na_V_1.5/QQQ structure revealed a high affinity binding site for propafenone in the central cavity. Propafenone engages both the Phe1762 and Tyr1769 (rat Na_V_1.5) on D_IV_ S6 helix by forming π-π stacking interaction and on-edge van der Waals interactions, respectively (Figure 3E). The positively-charged amino group blocks the exit of Na^+^ from the SF, which is similar to the piperidine of flecainide and the quinolone ring of quinidine (Jiang et al., 2020; Li et al., 2021a). The open activation gate of Na_V_1.5/QQQ is wide enough for propafenone passing through, which suggests that propafenone probably access its high affinity binding site through the open activation gate. In the resting state, the intracellular activation gate is closed, the antiarrhythmic and local anesthetic drugs may access their receptor sites through the fenestrations in a less efficient manner (Figure 3D) (Gamal El-Din et al., 2018; Catterall et al., 2020a), thus exhibiting low affinity blocking. Structural superposition of the Na_V_ isoforms show that the wall of the central cavity is nearly identical, suggesting it’s very difficult to achieve selective receptor site inside the central cavity. Thus, the state- and use-dependent block of the antiarrhythmic and local anesthetic drugs relies on the membrane potential and firing frequency (Hille, 1977; Hondeghem and Katzung, 1984), which allows the drugs to access their active sites in the favorable states without affecting the channels in unfavorable states (Catterall et al., 2020a).

### Fast Inactivation of Sodium Channel

Fast inactivation is one of the hallmark features of eukaryotic Na_V_ channels (Goldin, 2003). The fast inactivation of Na_V_ channels is crucial for preventing hyperexcitability and priming firing frequency (Catterall, 2000; Catterall et al., 2017). In 1973, Armstrong found that the fast inactivation was dramatically destructed by intracellularly applying proteolytic enzyme in the squid giant axons (Armstrong et al., 1973), indicating that the fast inactivation gate is located in the cytosol. Site-direct antibody studies identified that the fast inactivation gate is located in the loop between D_III_ and D_IV_ (Vassilev et al., 1988; Stühmer et al., 1989). More detailed point mutagenesis studies confirmed a triple-hydrophobic Ile-Phe-Met (IFM) motif in the D_III_-D_IV_ loop is responsible for the fast inactivation (West et al., 1992; Eaholtz et al., 1994). The cysteine substitution accessibility results indicated that the IFM-motif is buried in a receptor site inaccessible to solvent during inactivation process (Kellenberger et al., 1996). Further extensive site-directed mutagenesis screening studies mapped the IFM-motif receptor site which is constituted by the S4-S5 linkers of D_III_ and D_IV_, and also the cytoplasmic end of D_IV_-S6 helix (McPhee et al., 1994, 1998; Lerche et al., 1997; Smith and Goldin, 1997). Bases on these results, a hinged-lid mechanism for the fast inactivation was proposed, that is, the IFM-motif acts as a hydrophobic latch that binds to its receptor site to close the activation gate. Furthermore, the fast inactivation was found to be electromechanically coupled to the activation of the VSD_IV_ (Chanda and Bezanilla, 2002; Capes et al., 2013).

**FIGURE 4:**
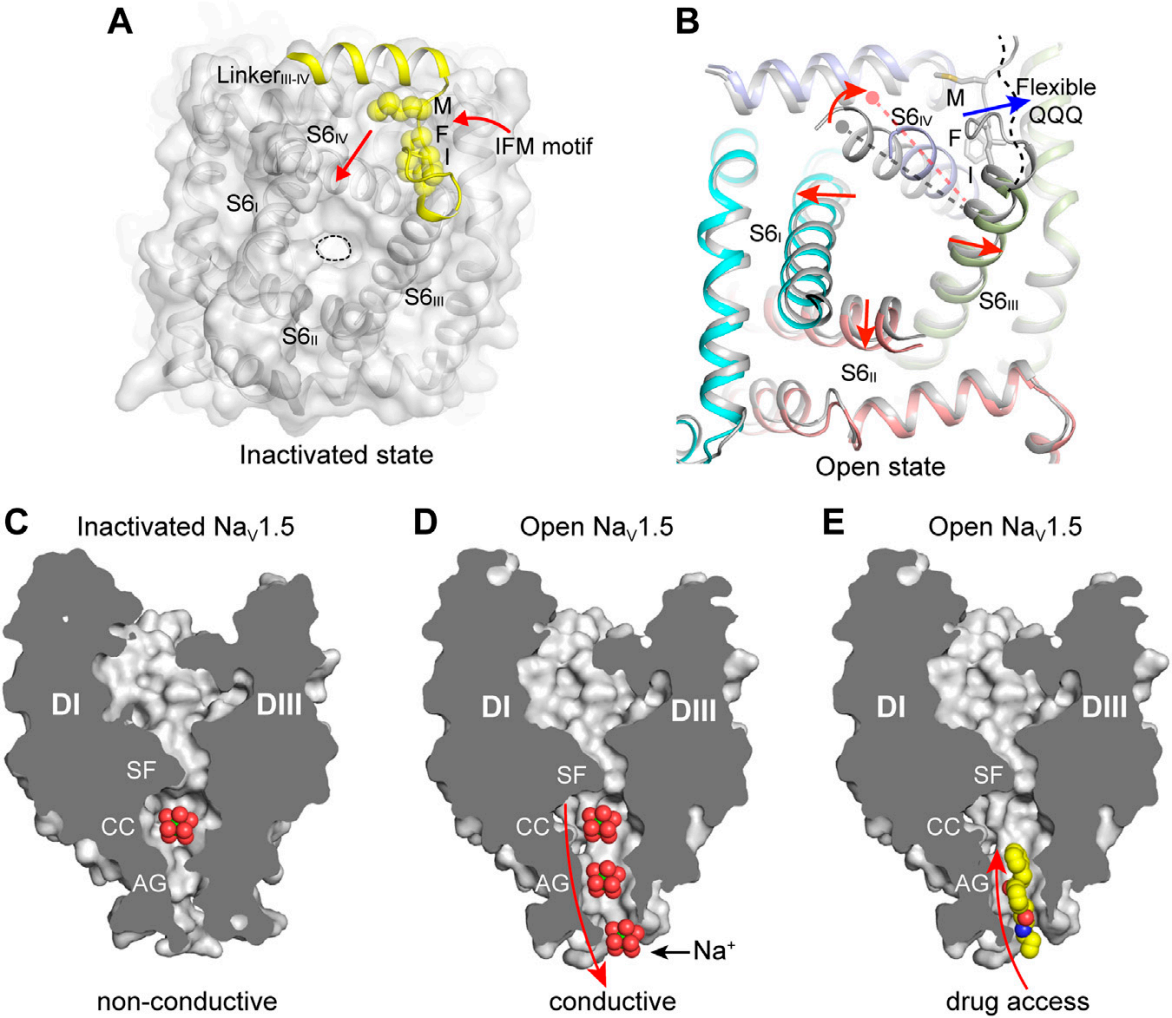
The IFM-motif mediated fast inactivation and the open activation gate. **(A)** Allosteric inhibition of mammalian Na_V_ channels by the IFM-motif (PDB code: 6J8I). The pore domain viewed from cytosol is shown in surface. The IFM-motif shown in yellow spheres. **(B)** Releasing of the IFM-motif leads to channel opening. Activation gate comparison between rat Na_V_1.5 (PDB code: 6UZ3) and Na_V_1.5/QQQ (PDB code: 7FBS). Red arrows indicate the S6 helix shift. **(C)** Non-conductive ion path of the inactivated state Na_V_ channel (PDB code: 6UZ3). **(D)** Na^+^ conductive ion path of the open-state Na_V_ channel (PDB code: 7FBS). **(E)** The open activation gate of Na_V_ channel provides potential accessing path for the open-state blockers. Propafenone is shown in spheres.

The solution structure of the D_III_ and D_IV_ linker showed that the flexible IFM-motif is tethered to an α-helix (Rohl et al., 1999), suggesting that the IFM-motif is readily available to bind to its receptor site once close to it. The detailed binding mode of the IFM-motif was consistently revealed by the cryo-EM structures of the eukaryotic Na_V_ channels (Jiang et al., 2020; Pan et al., 2019; Pan et al., 2018; Shen et al., 2019; Yan et al., 2017). In agreement with the mutagenesis studies and the hinged-lid model, the IFM-motif is embedded in a hydrophobic receptor site formed by the D_IV_-S6 helix and the S4-S5 linkers of D_III_ and D_IV_, adjacent to the intracellular activation gate (Figure 4A). The binding pose of the IFM-motif is further stabilized by extensive hydrophobic interactions and hydrogen-bond network (Jiang et al., 2020; Pan et al., 2018). In particular, the activated conformation of VSD_III_ and VSD_IV_ are required for the formation of the IFM-motif receptor site (Jiang et al., 2020). These structures also showed that the activation gate is in the non-conductive state with a diameter of <5 Å, elucidating an allosteric inhibition mechanism for the fast inactivation (Figures 2A, 4A). Meanwhile, the electromechanical coupling was observed to be disrupted by α-Scorpion toxins, which specifically bind to the VSD_IV_ and trap it in a deactivated state (Clairfeuille et al., 2019; Jiang et al., 2021c). There structures provide a structural framework for the fast inactivation of sodium channels. Pathogenic mutations target the fast inactivation gate and cause life-threatening diseases. For example, mapping the location of the arrhythmia associated mutations in Na_V_1.5 revealed that the gain-of-function mutations are dense around the fast inactivation gate (Jiang et al., 2020). These gain-of-function mutations impair the fast inactivation, thus cause the channel to generate large abnormal persistent currents or repetitive firing that lead to arrhythmias such as Long-QT syndrome type-3 (Clancy and Kass, 2005).

Sodium ions across the ion path are believed to undergo de-hydration by the SF and re-hydration in the central cavity, then pass the activation gate in hydrated form. The open state structures of BacNa_V_ channels have shown that the activation gate is wide enough for conducting hydrated Na^+^ (Lenaeus et al., 2017; Sula et al., 2017). The overall structure of the open state Na_V_1.5/QQQ is similar to the inactivated state Na_V_1.5 structures (Jiang et al., 2021a; Jiang et al., 2020). However, releasing of the IFM-motif from its receptor site caused marked local conformational changes in the activation gate (Figure 4B). Especially, The D_IV_-S6 helix shifts toward the receptor site by ∼6 Å at the cytoplasmic end. The resulting dilated activation gate of the Na_V_1.5/QQQ is sufficient for free passing of hydrated Na^+^, also provides an access for pore-blocking drugs such as propafenone (Figures 4C–E) (Jiang et al., 2021a).

### VSD and Gating Modifier Toxins

**FIGURE 5:**
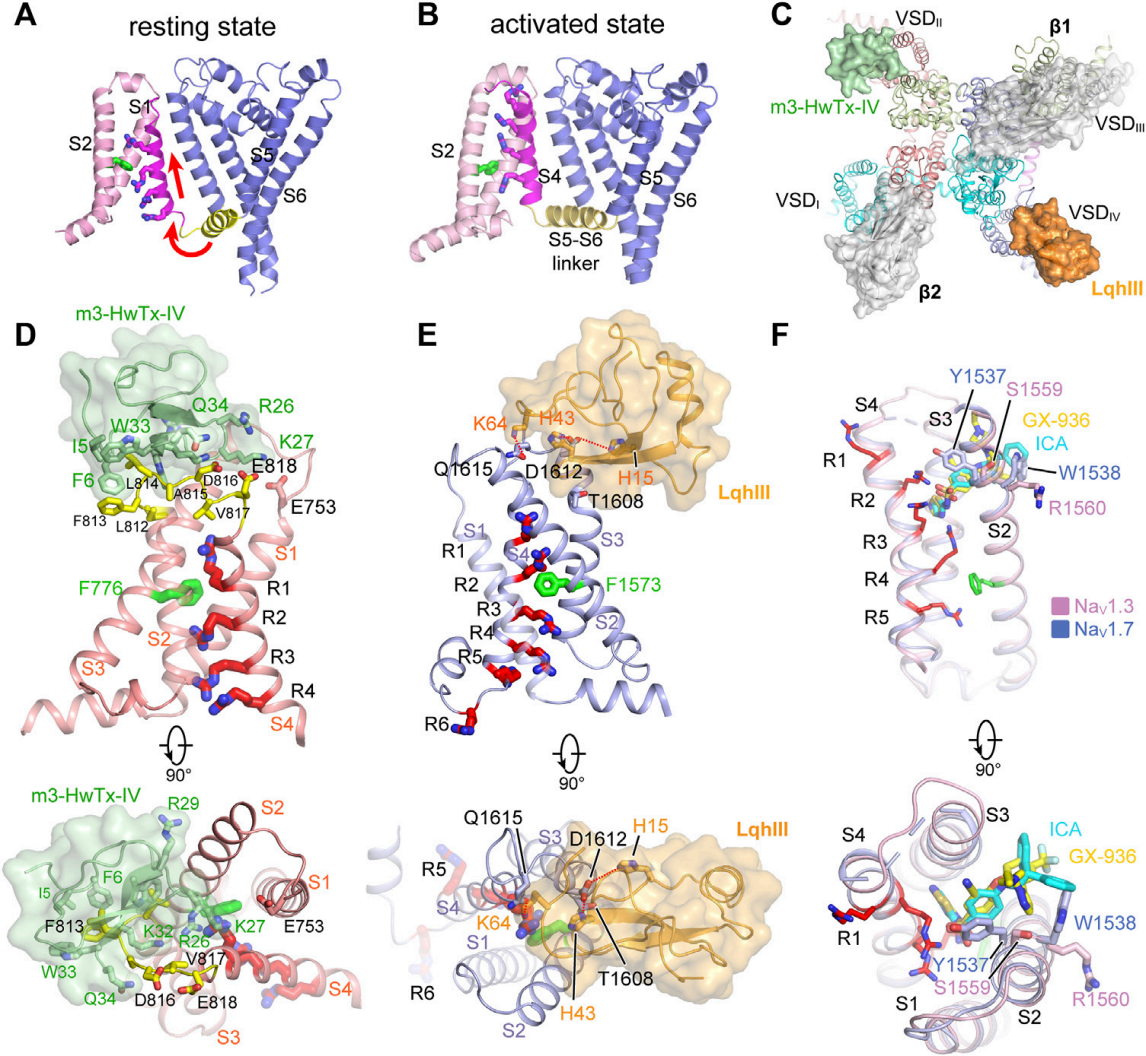
GMTs bind to VSDs of Na_V_ channels. **(A,B)** Resting (PDB code: 6P6W) and activated (PDB code: 3RVY) state structure of Na_V_Ab. Red arrows indicate conformational shifts between resting and activated state Na_V_Ab. The gating charges on the S4 helix shown side chains in sticks. **(C)** Site-3 toxin LqhIII (PDB code: 7K18) and site-4 toxin HwTxIV (PDB code: 7K48) bind to VSD_IV_ and VSD_II_ of Na_V_ channel, respectively. The Ig-like domain of β1 and β2 subunits project on the VSD_III_ and VSD_I_, respectively, which block the accessibility for potential GMTs binding to VSD_III_ and VSD_I_. **(D)** High affinity binding site for site-4 toxin HwTxIV in the deactivated VSD_II_ (PDB code: 7K48). **(E)** Detailed binding site for α-Scorpion toxin LqhIII in the deactivated VSD_IV_ (PDB code: 7K18). **(F)** Detailed binding site for aryl sulfonamide derivatives ICA121431 and GX-936 in the activated VSD_IV_ of Na_V_1.3 (PDB code: 7W7F) and Na_V_1.7 (PDB code: 5EK0), respectively.

The VSDs control both the activation and inactivation of the eukaryotic Na_V_ channels in response to membrane potential changes. The molecular gating mechanisms of Na_V_ channels have been reviewed by Ahern (Ahern et al., 2016) and Catterall (Catterall et al., 2017; 2020b). The Na_V_ channel amino acid sequence revealed that the fourth helix (S4) contains at least four positively-charged Arg or Lys (gating-charges) repeated in a pattern of every three-residues (Noda et al., 1984). In light of the sequence and the biophysical results, the “sliding-helix” and “helical-screw” models were soon proposed to explain the voltage sensing, both models suggest that the positive gating-charges on the S4 helix serve as voltage sensors which move up and down in the membrane in response to membrane potential changes (Catterall, 1986; Guy and Seetharamulu, 1986). The BacNa_V_ structures captured in the resting and activated states show that the gating-charges underwent a two helical-turns shift, supporting the “sliding-helix” or “helical-screw” models (Payandeh et al., 2011; Sula et al., 2017; Wisedchaisri et al., 2019; Zhang et al., 2012). Because there is no membrane potential in purification conditions (0 mV), most Na_V_ structures were determined in the activated state (Figures 5A,B). The S4 helix is wrapped by the S1-S3 helices, forming a V-shaped aqueous cleft toward the extracellular side. Three of the four gating-charges in the activated Na_V_Ab structure adopt the activated “up” conformation, which are neutralized by extracellular negatively-charged clusters (ENC). Wisedchaisri obtained the resting state structure of Na_V_Ab by a combination of importing positive voltage-shifting mutations and cysteine disulfide-bond lock (Wisedchaisri et al., 2019). Compared to the activated VSD, the VSD in the resting state shifts the S4 helix two helical-turns downward to the intracellular side, while the conformation of the S1-S3 helices remains unchanged (Figures 5A,B). The inward movement of the S4 helix further bends the S4-S5 linker helix *via* an elbow-like turn. The twisted S4-S5 linker directly causes the bending and rotation of the S6 helix to close the activation gate. These structures define a possible general mechanism for electromechanical coupling of voltage-gated ion channels.

Distinct from the homotetrameric BacNa_V_ channels, metazoan Na_V_ channels possess four non-identical VSDs (Noda et al., 1984; Catterall et al., 2017). The VSD_I_ and VSD_II_ have four gating-charges, VSD_III_ has five gating-charges, and VSD_IV_ has six to eight gating-charges. The four asymmetric VSDs response asynchronously to membrane depolarization (Chanda and Bezanilla, 2002; Ahern et al., 2016). This asynchronous activation is closely related to the voltage-dependent activation and fast inactivation of Na_V_ channels. According to the asynchronous gating model (Ahern et al., 2016), activation of VSD_I_-VSD_III_ precedes VSD_IV_ leading to channel opening, and VSD_IV_ activates subsequently to induce the fast inactivation (Chanda and Bezanilla, 2002; Chanda et al., 2004; Capes et al., 2013). The mammalian Na_V_ channel structures confirmed the asymmetric activation of the four VSDs (Pan et al., 2018; Pan et al., 2019; Shen et al., 2019; Jiang et al., 2020). The VSD_I_ and VSD_II_ closely resemble the activated VSD of Na_V_Ab, R1-R3 adopt the “up” conformation above the hydrophobic constriction stie (HCS). The VSD_III_ is more activated, K1-R4 point up and R5 is stuck in the HCS. In contrast, the VSD_IV_ is less activated, R1-R4 are located above the HCS, whereas R5-R6 point downward interacting with the intracellular negatively-charged clusters (INC). The VSD_III_ and VSD_IV_ also recover slower than the VSD_I_ and VSD_II_, which is probably because the VSD_III_ and VSD_IV_ carry more gating charges that limit their moving rate across the HCS (Capes et al., 2013; Goldschen-Ohm et al., 2013; Jiang et al., 2020).

### Modulation of Na_V_ Channels by Site-4 Toxins

**FIGURE 6:**
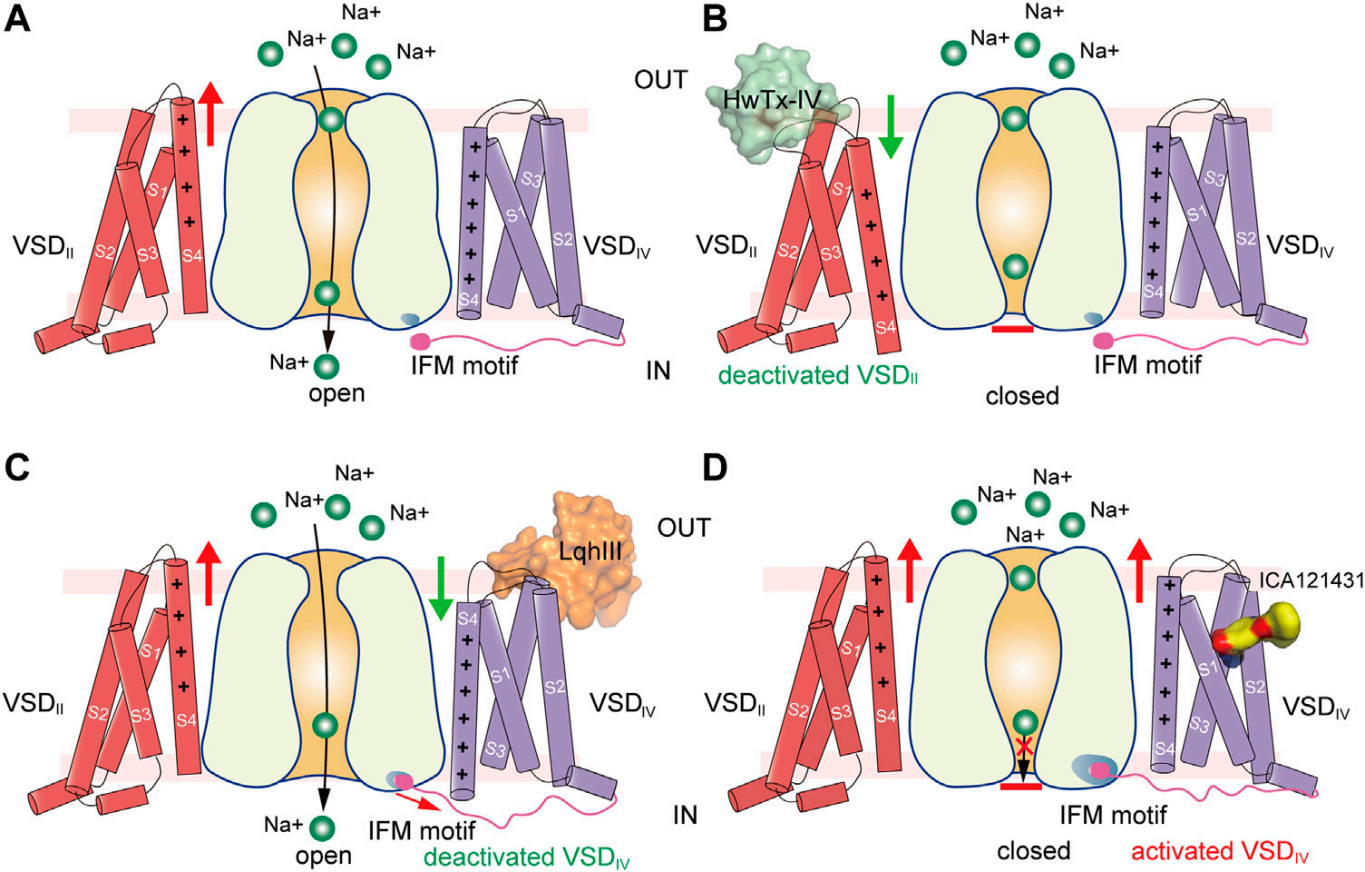
Putative models for modulation of VSDs of Na_V_ channels. **(A)** Activation of VSD_I_ and VSD_II_ cause channel opening. Green balls represent sodium ions. Red arrow indicates activation of the VSD. **(B)** Site-4 toxin HwTx-IV traps VSD_II_ in a deactivated state and inhibits channel opening. Green arrow indicates deactivation of the VSD. **(C)** α-Scorpion toxin LqhIII traps VSD_IV_ in a deactivated state and inhibits fast inactivation. **(D)** Aryl sulfonamide analogue ICA121431 binds to activated VSD_IV_ and inhibits channel opening.

Many natural gating modifier toxins (GMTs) target the VSDs and alter Na_V_ channel properties (Catterall et al., 2007). These GMTs are important tools to study the Na_V_ channel properties because of their high-affinity and specific binding mode (Lazdunski et al., 1986). Among them, a group of polypeptide toxins from spiders or scorpions specifically bind to the VSD_II_ of Na_V_ channels, classified as site-4 neurotoxins, which are used as weapons to paralyze prey (Catterall et al., 2007; Dufton and Rochat, 1984; Rochat et al., 1979). The site-4 toxins were found to inhibit the activation of Na_V_ channels (Sokolov et al., 2008; Xiao et al., 2008), or cause abnormal activation (Cestèle et al., 1998). The binding site for the site-4 toxins is located in the VSD_II_, especially the extracellular loops linking S1-S2 and S3-S4 (Cestèle et al., 1998; Marcotte et al., 1997). Two site-4 toxins, Protoxin-II (β/ω-theraphotoxin-Tp2a; ProTx-II) from the Peruvian green velvet tarantula *Thrixopelma pruriens* (Middleton et al., 2002) and Huwentoxin-IV (μ-theraphotoxin-Hs2a; HwTx-IV) from the Chinese bird tarantula *Haplopelma schmidti* (Peng et al., 2002), show higher potency in inhibiting Na_V_1.7 than other Na_V_ isoforms. Shen reported the cryo-EM structures of human Na_V_1.7 in complex with ProTx-II and HwTx-IV, showing that ProTx-II binds to both the VSD_II_ and VSD_IV_, whereas HwTx-IV only binds to the VSD_II_ (Figure 5C) (Shen et al., 2019). However, the EM density for ProTx-II and HwTx-IV are insufficient to define a detailed binding site. The binding of the two toxins appear only to induce subtle local conformational changes in the activated VSDs (Shen et al., 2019). It has been shown that ProTx-II has higher affinity to the resting state of Na_V_1.7 than the activated state (Sokolov et al., 2008; Xu et al., 2019). These observations suggest that the binding poses of ProTx-II and HwTx-IV in the Na_V_1.7 structures may reflect a low-affinity binding state. Meanwhile, Xu reported the ProTx-II bound structures of a chimeric bacterial Na_V_Ab with VSD_II_ of Na_V_1.7 (designated as Na_V_Ab-Na_V_1.7VSD_II_) in different activation states (Xu et al., 2019). By sorting cryo-EM images of the Na_V_Ab-Na_V_1.7VSD_II_-ProTxII yielded two maps in distinct conformations. The VSDs of the major class are in the activated conformation, while the structure of the minor class clearly showed that ProTx-II binding shifted the S4 helix ∼10 Å downward into a deactivated state. The deactivated Na_V_Ab-Na_V_1.7VSD_II_-ProTxII may represent the high-affinity binding state for ProTx-II. However, the limited resolution prevented revealing a clearer picture for the high-affinity binding site (Xu et al., 2019). Subsequently, Wisedchaisri refined the chimeric Na_V_Ab-Na_V_1.7VS2A construct by importing a voltage-shifting mutation to stabilize the channel in the resting state even under positive membrane potential (Wisedchaisri et al., 2021), and reported the structure of the Na_V_Ab-Na_V_1.7VS2A complexed with a modified HwTx-IV (m3-HwTx-IV) (Rahnama et al., 2017; Revell et al., 2013). The Na_V_Ab-Na_V_1.7VS2A:m3-HwTx-IV structure unveiled the high affinity binding site for m3-HwTx-IV and demonstrated that the m3-HwTx-IV inhibits the channel by locking the VSD_II_ in the resting state (Wisedchaisri et al., 2021). The m3-HwTx-IV forms extensive interactions with the S3-S4 loop (_811_ELFLADVE_818_) of the VSD_II_, which is consistent with the deactivated Na_V_Ab-Na_V_1.7VSD_II_-ProTxII structure (Xu et al., 2019) and the site-direct mutagenesis studies (Xiao et al., 2008; Xiao et al., 2010). Among the S3-S4 loop (_811_ELFLADVE_818_) of the VSD_II_, the F813G mutation drops the affinity of ProTx-II by ∼9-fold, but does not affect the binding of HwTx-IV (Xiao et al., 2010); by contrast, the E818C mutation significantly increases the affinity of HwTx-IV, whereas it has little effect on ProTx-II (Xiao et al., 2010; Xu et al., 2019). These opposite effects suggest that the detailed binding poses for the HwTx-IV and ProTx-II are different, despite both of the toxins bind to the same region of the VSD_II_ (Figure 5D). The Na_V_Ab-Na_V_1.7VSD_II_-ProTxII and Na_V_Ab-Na_V_1.7VS2A:m3-HwTx-IV structures elucidate a common inhibition mechanism for the site-4 toxins (Figures 6A,B), that is, the positively-charged residues in the C-terminus of the toxins insert their side-chains into the cleft of the VSD_II_ and trap the VSD_II_ in the resting state to prevent the activation (Xu et al., 2019; Wisedchaisri et al., 2021).

The site-4 toxins ProTxII and HwTx-IV are more potent in inhibiting Na_V_1.7 than other Na_V_ subtypes (Schmalhofer et al., 2008). Na_V_1.7 plays crucial roles in pain sensation (Bennett et al., 2019; Dib-Hajj and Waxman, 2019), thus, selective inhibition of Na_V_1.7 may bring potentially non-addictive medical benefits in treating chronic pain. Given the fact that Na_V_1.7 are in the resting state for most of the time in the nociceptive afferents (Wisedchaisri et al., 2021), the resting-state trapping mechanism revealed by the above structures suggests that optimization of these gating-modifier toxins could potentially generate effective and selective analgesics. These structures also provide structural templates for future development of new analgesics targeting the resting-state Na_V_ channels.

### Modulation of Na_V_ Channel by α-Scorpion Toxins

α-Scorpion toxins belong to a family of peptide neurotoxins which inhibit fast inactivation of Na_V_ channels, causing prolonged and/or repetitive action potentials (Catterall et al., 2007; Jiang et al., 2021c; Kopeyan et al., 1974). Site-directed mutagenesis studies have localized the binding site of α-Scorpion toxins in the S3-S4 loop of VSD_IV_, termed site-3 (Rogers et al., 1996; Thomsen and Catterall, 1989). In addition, a negatively-charged Asp or Glu residue on S3 of VSD_IV_ was identified as the key determinant for the α-Scorpion toxins binding (Benzinger et al., 1998; Rogers et al., 1996). These findings also support the electromechanical coupling between the fast inactivation gate and the VSD_IV_. The α-Scorpion toxin LqhIII and AaHII are lethal toxins extracted from venoms of the “deathstalker scorpion” *Leiurus quinquestriatus hebraeus* and the *Androctonus australis Hector* “man killer” scorpion (Chen and Heinemann, 2001; Martin et al., 1987), both of which dramatically inhibit the fast inactivation of the Na_V_ channels. In particular, LqhIII exhibits higher affinity to Na_V_1.5 and extremely slow disassociation rate (Chen and Heinemann, 2001). The cryo-EM structures of rat Na_V_1.5-LqhIII and the chimeric Na_V_Pas-Na_V_1.7VSD_IV_-AaHII reveal detailed binding site for the α-Scorpion toxins (Clairfeuille et al., 2019; Jiang et al., 2021c). Consistent with previous mutagenesis studies, LqhIII and AaHII engage the S3-S4 loop of VSD_IV_ by forming broad interaction interface (Figure 5C). The D1612 in D_IV_-S3 of Na_V_1.5 and D1586 in D_IV_-S3 of Na_V_1.7 form critical polar interactions with LqhIII and AaHII, respectively. The toxins binding shifts the S4 helix about two helical-turns inward and traps the VSD_IV_ in an intermediate activated state (Figure 5E). The relatively flexible positively-charged C-terminal tails of LqhIII and AaHII dock into the extracellular aqueous cleft of VSD_IV_, displacing the gating-charges and preventing them moving upward. As expected, the two structures show very similar overall binding sites for the α-Scorpion toxins, and also similar conformational shifts in the VSD_IV_ induced the toxins binding (Clairfeuille et al., 2019; Jiang et al., 2021c). Furthermore, the Na_V_1.5-LqhIII structure reveals a common gating mechanism for the α-Scorpion toxins (Figure 6C). That is, the α-Scorpion toxins specifically recognize the high-affinity binding site in the VSD_IV_ and trap the VSD_IV_ in the intermediate activated state, then deactivation of the VSD_IV_ destabilizes the fast inactivation gate thus favors the channel opening (Jiang et al., 2021c).

Although the site-3 toxins and site-4 toxins bind to distinct receptor sites and cause different effects on Na_V_ channels, these two types of neurotoxins use similar VSD trapping mechanism to modulation channel gating (Figures 5D,E). The different modulation effects are because that the activation and fast inactivation of Na_V_ channels rely on the activation of VSD_I_-VSD_II_ and VSD_IV_, respectively (Chanda and Bezanilla, 2002). From the Na_V_1.4-β1, Na_V_1.7-β1-β2, Na_V_1.2-β2, Na_V_1.1-β4 and Na_V_1.3-β1-β2 complex structures, we have known that β1 subunit projects its Ig-like domain on the VSD_III_, β2 and β4 project their Ig-like domain on the VSD_I_, respectively (Figures 1B, 5C). Consequently, the accessibility of VSD_III_ and VSD_I_ for potential neurotoxins are blocked by the β subunits, which may explain that only a few neurotoxins were reported to bind to VSD_III_ or VSD_I_. For instance, Hm-3, from the crab spider *Heriaeus melloteei*, was reported to inhibit Na_V_1.4 by binding to the VSD_I_ with micromolar affinity (Männikkö et al., 2018). Recently, a spider toxin Gr4b from *Grammostola rosea* appears to selectively impair fast inactivation of Na_V_1.9 by binding to its VSD_III_ (Peng et al., 2021). However, the detailed binding sites and the underlying mechanisms for those toxins need further investigation. Meanwhile, because the β subunits bind loosely to Na_V_1.5 and Na_V_1.8 (Jiang et al., 2020), this raises a possibility that candidate modulators such as engineered neurotoxins or nanobodies can target Na_V_1.5 or Na_V_1.8 by binding to the VSD_III_ or VSD_I_ without affecting other β subunit-bound Na_V_ isoforms.

### Aryl Sulfonamides Selectively Binds to VSD_IV_

The apo-form Na_V_ channel structures show that activation of the VSD_IV_ tightens the fast inactivation gate to close the channel (Jiang et al., 2020; Pan et al., 2018; Yan et al., 2017). Thus, trapping the VSD_IV_ in the activated conformation inhibits the opening of Na_V_ channels. In agreement with this concept, a family of synthetic aryl sulfonamide derivatives exhibit potent and selective inhibition of Na_V_ channel isoforms *via* binding to the VSD_IV_ (McCormack et al., 2013). For instance, PF-04856264 and GX-936 selectively and potently inhibit Na_V_1.7 (Ahuja et al., 2015; McCormack et al., 2013), whereas ICA121431 exhibits potent inhibition of Na_V_1.3/Na_V_1.1 with a factor of >1000-fold over other isoforms (McCormack et al., 2013). The promising selective inhibition is of great interest for developing potentially non-addictive analgesics (Alsaloum et al., 2020). The crystal structure of the chimeric Na_V_Ab-Na_V_1.7VSD_IV_ in complex with GX-936 revealed the first binding site for the aryl sulfonamide analogues (Ahuja et al., 2015). The GX-936 sticks deep into the extracellular aqueous cleft of the VSD_IV_, and its negatively-charged warhead engages the fourth gating-charge (R4), locking the VSD_IV_ in the activated conformation (Figure 5F). Subsequently, the complex structure of human Na_V_1.3/β1/β2-ICA121431 confirmed the conserved binding site for the aryl sulfonamide analogues (Li et al., 2022). The conserved warhead of the aryl sulfonamide derivatives determines the potency *via* strong electrostatic interactions with the positively-charges R4, which is supported by the R4A mutation dramatically decreasing the affinity of GX-936 by >2000-fold (Ahuja et al., 2015). In addition, the S1559/R1560 on S2 helix of the Na_V_1.3-VSD_IV_ are responsible for recognizing the tail of ICA121431, and Y1537/W1538 at the equivalent position on Na_V_1.7 are more favorable for GX-936 binding (Ahuja et al., 2015; Li et al., 2022; McCormack et al., 2013). Superposition of the ICA121431 bound Na_V_1.3-VSD_IV_ with the deactivated LqhIII bound Na_V_1.5-VSD_IV_ shows that the gating charges in the deactivated VSD_IV_ cannot form proper interactions with the anionic warhead, elucidating why the aryl sulfonamide derivatives are in favor of binding to the activated VSD_IV_. In the Na_V_1.3/β1/β2-ICA121431 structure, the fast inactivation gate binds tightly to its receptor site resulting in a non-conductive activation gate (Figure 6D), which provides a full-picture for understanding the allosteric inhibition mechanism of the aryl sulfonamide antagonists (Li et al., 2022).

Several aryl sulfonamide derivatives, such as PF-05089771, GDC-0276 and RG6029, which selectively inhibit Na_V_1.7 (Alexandrou et al., 2016; McDonnell et al., 2018; Rothenberg et al., 2019; Alsaloum et al., 2020), have failed in Phase I or Phase II clinical trials because of low efficacy (Kingwell, 2019; Alsaloum et al., 2020). These discouraging results suggest that isoform selectivity is not the only challenge for developing candidate analgesics targeting pain related Na_V_ channels.

## Summary and Prospects

Na_V_ channels play fundamental roles in electrical signaling. Extensive studies on the biophysical characterization, gene sequence, physiological functions, ligands modulation and pharmacology of Na_V_ channels have greatly enriched the knowledge of Na_V_ channels. During the last a few years, cryo-EM structures of Na_V_ channels from nerve, cardiomyocytes and skeletal muscle were resolved in different functional states or with the binding of distinct modulators. These structures provide in-depth mechanistic insights into the architecture, activation, inactivation, ion selectivity, electromechanical coupling, ligand modulation, and structural pharmacology of Na_V_ channels. The structure-based drug design will be accelerated by those structural studies, which could potentially generate more efficient, safer and selective drugs for the treatment of Na_V_ channel associated diseases. The cryo-EM technique will have broader application prospects in validating the structural pharmacology of Na_V_ channels, also in expanding the fundamental understanding of Na_V_ channel structure and function. We anticipate that more mammalian Na_V_ channel structures in different functional states including the resting-state will be achieved, and high-resolution structures will also be very important to unveil more detailed information such as ion selectivity and lipid-channel interactions.
